# Classification-Biased Apparent Brain Age for the Prediction of Alzheimer's Disease

**DOI:** 10.3389/fnins.2021.673120

**Published:** 2021-05-28

**Authors:** Ali Varzandian, Miguel Angel Sanchez Razo, Michael Richard Sanders, Akhila Atmakuru, Giuseppe Di Fatta

**Affiliations:** Department of Computer Science, University of Reading, Reading, United Kingdom

**Keywords:** Alzheimer's disease, brain age, magnetic resonance imaging, machine learning, predictive and descriptive models, explainable artificial intelligence

## Abstract

Machine Learning methods are often adopted to infer useful biomarkers for the early diagnosis of many neurodegenerative diseases and, in general, of neuroanatomical ageing. Some of these methods estimate the subject age from morphological brain data, which is then indicated as “brain age”. The difference between such a predicted brain age and the actual chronological age of a subject can be used as an indication of a pathological deviation from normal brain ageing. An important use of the brain age model as biomarker is the prediction of Alzheimer's disease (AD) from structural Magnetic Resonance Imaging (MRI). Many different machine learning approaches have been applied to this specific predictive task, some of which have achieved high accuracy at the expense of the descriptiveness of the model. This work investigates an appropriate combination of data science techniques and linear models to provide, at the same time, high accuracy and good descriptiveness. The proposed method is based on a data workflow that include typical data science methods, such as outliers detection, feature selection, linear regression, and logistic regression. In particular, a novel inductive bias is introduced in the regression model, which is aimed at improving the accuracy and the specificity of the classification task. The method is compared to other machine learning approaches for AD classification based on morphological brain data with and without the use of the brain age, including Support Vector Machines and Deep Neural Networks. This study adopts brain MRI scans of 1, 901 subjects which have been acquired from three repositories (ADNI, AIBL, and IXI). A predictive model based only on the proposed apparent brain age and the chronological age has an accuracy of 88% and 92%, respectively, for male and female subjects, in a repeated cross-validation analysis, thus achieving a comparable or superior performance than state of the art machine learning methods. The advantage of the proposed method is that it maintains the morphological semantics of the input space throughout the regression and classification tasks. The accurate predictive model is also highly descriptive and can be used to generate potentially useful insights on the predictions.

## 1. Introduction

Alzheimer's disease (AD) is a terminal neurodegenerative disease and the most common type of dementia. The number of people diagnosed with AD is anticipated to go up during the coming decades, in a way that by 2050 more than 1.5% of the world's population are estimated to have AD (Brookmeyer et al., 2007; Crous-Bou et al., 2017).

Although the definitive diagnosis of AD is only possible at the brain autopsy after death (Blennow et al., 2006), diagnosis of AD in living subjects can be achieved with the help of biomarkers obtained from brain-imaging technologies such as magnetic resonance imaging (MRI), computerised tomography (CT), and positron emission tomography (PET). There is no single diagnostic test for AD. The Mini-Mental State Exam (MMSE) is commonly used as assessment for mild cognitive impairment (MCI), which is considered a high risk factor to develop AD. The MMSE is easy to administer and used for screening. However, the test has a high false negative rate.

It is believed that AD pathophysiological process starts many years, or even decades, before any evident cognitive decline and the onset of clinical dementia (Budson and Solomon, 2012). Early diagnosis and early intervention are extremely important in order to contain the significant impact in terms of human, social, and economical costs. However, the potential benefits of an early diagnosis are matched by its difficulty due to a long asymptomatic stage and the lack of definitive biomarkers.

To aid with the diagnosis of AD, the importance of brain structural magnetic resonance imaging has been recognised due to its ability to unveil atrophy in different regions of the brain (Fox and Schott, 2004). However, manual evaluation and measurement of different regions of the brain from MRI scans (Jack et al., 1992) do not capture the whole scale of the atrophy and is time-consuming.

Imaging biomarkers to help the diagnosis and the investigation of neurodegenerative disorders are receiving an increasing attention (Young et al., 2020). In particular, the adoption of machine learning algorithms provides the opportunity to generate useful insights and potentially accurate tools, thanks to the availability of larger multi-source data sets (Bron et al., 2015). Support Vector Machines (SVM) are an attractive solution for applications in many scientific domains that require a supervised analysis of large data sets in large and sparse feature spaces. PCA and its derivatives have been another very popular approach to deal with high-dimensional domains, such as brain images and morphological data. More recently, Deep Neural Networks (DNN) have also become increasingly popular for the analysis of brain MR images because of their successful applicability to image processing and, in general, to dealing with predictive problems in high-dimensional domains.

Several approaches have proposed the use of SVM and DNN on brain images and on the morphological data extracted from them to classify AD (Lao et al., 2004; Fan et al., 2005; Mourão-Miranda et al., 2005; Kawasaki et al., 2007; Kloppel et al., 2008). Although these methods can help dealing with the curse of dimensionality and may achieve a high classification accuracy, they are black-box approaches that lack descriptiveness: their models are particularly difficult to interpret and do not help in providing an explanation behind the classification predictions.

For the aim of providing both predictive accuracy and descriptiveness in the classification task, this work investigates a combination of machine learning algorithms to estimate and use a new feature referred to as Apparent Brain Age (ABA), which is biased to the specific classification task. In this case, ABA is inferred to be specifically predictive of AD.

Although in this work the focus is on AD classification, the aim of a contextual specificity is more general: improving the predictive power of the estimated brain age for a specific pathology would allow to develop an ensemble of discriminative ABA models for a group of neurodegenerative diseases.

The data set adopted in this work consists of brain T1-weighted structural MRI scans from 1,901 subjects retrieved from three publicly available repositories. In the experimental analysis the proposed method is compared with other machine learning algorithms, such as SVM and DNN. Various configurations of the proposed workflow are considered to highlight the relative contribution of different components.

The contributions of this work are briefly summarised and consist of:

- the introduction of a goal-conditioned brain age estimation, the Apparent Brain Age (ABA),
- the adoption of an inductive bias based on a feature selection technique in order to improve the classification accuracy of the estimated brain age,
- the design of a data workflow with a combination of only linear models to preserve the original input space semantics,
- the definition of a feature score to directly measure the specific contribution of each selected morphological region to the classification prediction,
- the presentation of a rigorous experimental comparative analysis to validate the method and to show it can achieve comparable or superior accuracy than state of the art machine learning methods, and
- the presentation of test cases to demonstrate the applicability of the classification method and its explainability approach.

The rest of the paper is organised as follows. Section 2 briefly discusses some related work on brain age estimation. Section 3 systematically presents the proposed method, including the description of the data acquisition and pre-processing, the design of the general data workflow, the machine learning components, and the definition of the feature score associated to the classification task. Section 4 presents the experimental analysis, discusses the main results and analyses a few test cases to demonstrate the model explainability. Section 5 provides some final discussion with a direct performance comparison with recent approaches based on the same data sources. Finally, section 6 provides some general conclusions and future research directions.

## 2. Related Work

Relevant work on brain age estimation has investigated regression models built on healthy control subjects to detect abnormal aging under neurodegenerative conditions. The most notable example is the Brain Age Gap Estimation (BrainAGE) (Franke et al., 2010; Franke and Gaser, 2019). The BrainAGE is then used as a biomarker to predict the progression of patients from MCI to AD in Gaser et al. (2013) and to classify AD in Franke and Gaser (2014). BrainAGE is inferred by means of Relevance Vector Regression (RVR) and Support Vector Regression (SVR) to build the age regression model of healthy subjects based on Voxel-Based Morphometry (VBM) after applying PCA to 3, 700 voxels to reduce the dimensionality of the input space.

In addition to BrainAGE, there have been other studies on brain age estimation, where, similarly to BrainAGE, the optimisation strategy of the model is minimising the age regression residuals and maximising the correlation between estimated age and actual age in healthy control subjects. The Brain Estimated Age Difference (Brain-EAD) proposed by Beheshti et al. (2020) implements a similar approach to BrainAGE on AD and Parkinson's Disease (PD) using SVR to build the Brain-EAD model. Deep Brain Network (DeepBrainNet) (Bashyam et al., 2020) estimates the brain age using Deep Neural Networks on a relatively large number of subjects (11,729) spanning over multiple sites and studies with the aim to classify multiple diseases (AD, Schizophrenia, Mild Cognitive Impairment, and Depression).

In this work, a brain age estimation approach similar to BrainAGE, Brain-AED, and DeepBrainNet is adopted. However, an important difference is that the proposed estimate of the brain age is goal-conditioned: the proposed Apparent Brain Age (ABA) is inferred from a subset of features that are selected with a method biased toward the specific classification task. ABA is not the estimation of the biological age of the entire brain, rather of an automatically selected subset of morphological regions, which result being highly predictive for the specific classification task.

The brain age estimation model proposed in this work is not only aimed at maximising the correlation with the chronological age for healthy subjects, which can then be adopted as a general indication of a synchrony or a gap between chronological and biological age of the whole brain. The ABA model is attempting to estimate the biological age of a few morphological regions, which are highly predictive of AD and are automatically selected. This is a fundamental difference to the common brain age estimation models such as BrainAGE, Brain-AED, and DeepBrainNet. The model proposed in this paper aims at maximising the classification accuracy while inferring the ABA model, therefore it is biased toward the classification of the specific neurodegenerative disease.

## 3. Method

**Figure 1:**
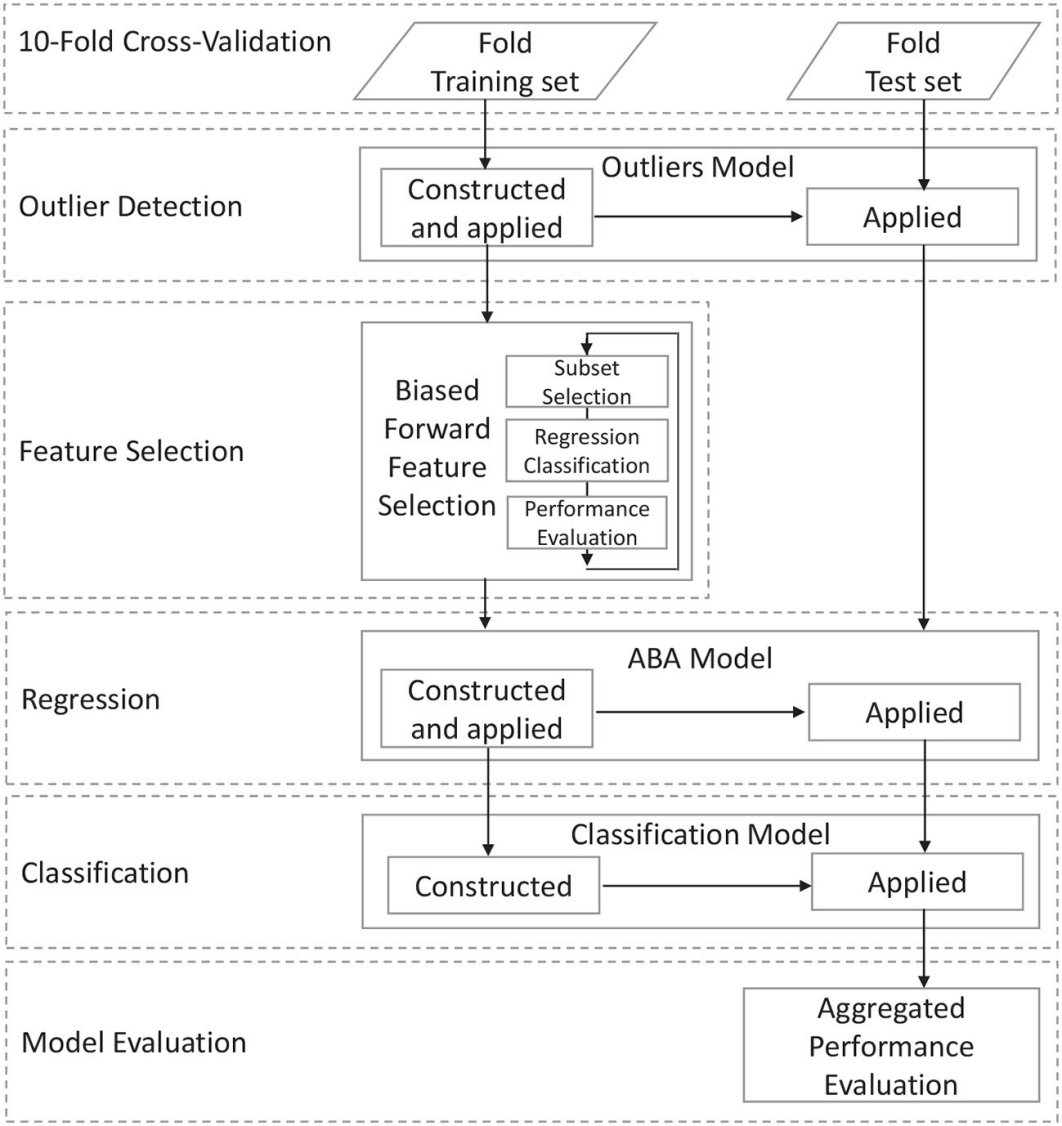
Overall data workflow of the proposed method.

The classification task is performed on morphological data extracted from structural MR brain images. After an initial data acquisition and image pre-processing task, the general data processing workflow is shown in Figure 1 and consists of a number of steps that address specific aspects of the processing pipeline aimed at achieving high accuracy and high descriptiveness of the predictive model. The first step requires the definition of the input data and the partitioning into training and test subset for the specific performance estimation methodology. This step includes data acquisition, pre-processing, and cleansing, which is described in details in the next section.

### 3.1. Data Acquisition and Pre-processing

The data set adopted in this work consists of brain T1-weighted structural MRI scans with slice thickness of 1.5 mm from 1, 901 subjects retrieved from three publicly available repositories, the Alzheimer's Disease Neuroimaging Initiative (ADNI), the Australian Imaging Biomarker & Lifestyle Flagship Study of Ageing (AIBL), and the Information eXtraction from Images (IXI).

The selected ADNI data include 390 subjects with an AD diagnosis and 715 cognitively normal subjects (CN). The selected AIBL data include 79 subjects with an AD diagnosis and 484 cognitively normal subjects (CN). The selected IXI data include 233 control subjects (CN) in an age range similar to the ADNI and AIBL data. The inclusion of the IXI data is useful as it allows for a larger number of normal control subjects and from different sources.

Note that the ADNI repository contains multiple images for the same subject over a few studies. The adopted images were selected among the screening and baseline scans as those were the earliest available for a subject. This is motivated by the goal of providing a diagnostic tool for early diagnosis. Where multiple images were available for the same subject at the same time, the image resulting with the highest contrast-to-noise ratio (CNR) is selected.

ADNI and AIBL participants have an age range of 55–90 with CN and AD diagnosis, whereas IXI has younger subjects with only CN diagnosis. To use the data from IXI, the subjects with age range comparable and consistent to ADNI and AIBL are selected.

Table 1 provides the distribution of subjects adopted in the experimental analysis.

**Table 1:** Distribution of the 1,901 subjects adopted in this study.

				**Age**			
**Gender**	**Source**	**Group**	**Number of subjects**	**Mean**	**SD**	**Min**	**Max**
Male	ADNI	AD	213	75.82	7.86	55.3	90.4
	ADNI	CN	317	74.2	6.36	56.2	90.3
	AIBL	AD	34	74.65	9.07	58	89.4
	AIBL	CN	207	74.36	7.83	54.6	89.8
	IXI	CN	90	65.5	7.27	55.09	86.2
Female	ADNI	AD	177	74.29	8.07	55.2	91
	ADNI	CN	398	72.1	6.24	55.6	89.9
	AIBL	AD	45	75.27	7.81	56.3	88.4
	AIBL	CN	277	74.44	7.31	55.2	88
	IXI	CN	143	65.1	6.31	55.22	86.32

All the images were pre-processed with FreeSurfer version 6.0 (Fischl et al., 2002) to carry out operations such as skullstripping, image registration, cortical and subcortical segmentation, hippocampal subfields segmentation, estimation of cortical thickness, surface and volume.

The pre-processing step generates a large set of files with numerical measurements associated to specific region of interests (ROI). The data generated by the pre-processing is extracted, filtered, and cleaned with KNIME (Berthold et al., 2006) and its extension KSurfer (Sarica et al., 2014a).

The total number of features extracted from the data generated by FreeSurfer is 446. The estimated total intracranial volume (ICV) is not included and ICV normalisation is not carried out. During the data cleaning step, a total of 33 features are removed due to containing errors or being duplicates. In the reminder the numerical brain measurements are referred to as features *F* = {*f*_*i*_}, where |*F*| = 413. No domain-specific knowledge is used to apply any filter to the features.

During testing, a Standardisation (z-score normalisation) model is computed on the training data partition and applied to the input features *F* of both training and test partitions.

The input data to the processing described in the next sections is the feature set *F*, the chronological age (age), the gender, and the classification group (AD, CN) of the subjects. The analysis has been carried out for each gender group separately as many studies, e.g. (Ritchie et al., 2018), have reported gender differences in the brain structure, though specific regional patterns and their relevance are not completely clear yet.

### 3.2. Outlier Detection

Outliers are input data records that are unexplainable and different from the rest of the data. These may be caused by head movements of the subject during the scan, malfunctions of the medical equipments or natural variability of human brain structures. For example, there is evidence of greater male variability in regional brain structures (Wierenga et al., 2020). Some of these outliers can be easily filtered with an analysis of the contrast-to-noise ratio (CNR) after the pre-processing carried out with FreeSurfer. Others may require an explicit detection and filtering process.

An important consideration is whether the specific machine learning algorithm is able to cope with outliers. For example, SVM have the ability to identify and implicitly handle outliers, while simple models like linear regression inference can be particularly sensitive to the presence of outliers. The proposed approach is intentionally using the simplest possible models to preserve model explainability and consequently requires the adoption of an explicit outliers detection step.

Removing outliers helps avoiding generating skewed models, but it also reduces the available input data. Therefore there should be a trade-off between the likelihood that some instances are outliers (how different they are from the rest of the data) and the number of outliers to be removed.

In the selection of the outlier detection (OD) technique, the number of features (|*F*|) is an important factor as some methods such as Local Outlier Filter (LOF) (Breunig et al., 2000) are only efficient at detecting outliers in a low dimensional data set. In this case, a high dimensional OD method is required. Among high dimensional OD methods, Isolation Forest (iForest) (Liu et al., 2008) and Angle-based Outlier Detection (ABOD) (Kriegel et al., 2008) are regarded as two of the best OD methods (Domingues et al., 2018), where iForest has a much lower computational complexity and is adopted in this work.

iForest is a tree-based outlier detection technique which uses random forests. It does not perform the profiling of normal instances (inliers) in order to avoid false positives (identifying normal instances as outliers). Outliers are detected based on the fact that they are "few and different," therefore it isolates outliers rather than profiling inliers.

In an iForest model, there are three main hyper-parameters: sub-sampling size ψ, height limit *l*, and number of iTrees *t*. Following the literature recommendations as well as a preliminary analysis of the data, the following parameters are adopted: ψ = 256, *l* = 8, and *t* = 100.

**Figure 2:**
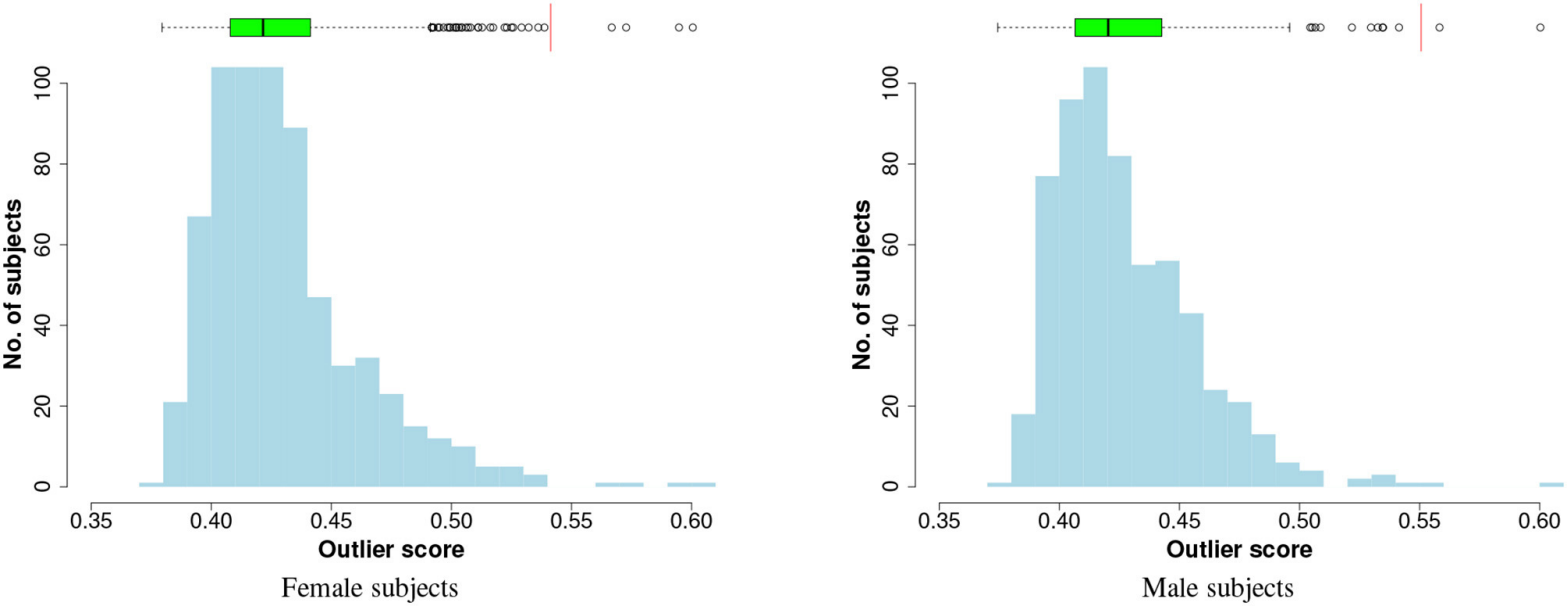
Histogram and box plot of iForest outlier scores. The red vertical lines on the boxplots are the cutoff thresholds that identify four and two outliers, respectively, in female and male subjects.

To visualise the results of outlier detection, the iForest technique is applied to each gender group separately to generate outliers scores in an unsupervised way (the class labels are not used). When the method is applied to the entire input data, the distributions of the outlier scores are plotted in Figure 2, which shows that the scores follow a right-skewed (positively-skewed) distribution with a tail: a few outlier instances can be identified in the tails. To select the cut-off point, the Tukey's method (Salgado et al., 2016) is used:

(1) $$\begin{array}{r} cutoff=Q3+3\cdot IQR, \end{array}$$

where Q3 and IQR are third quartile and inter-quartile range in the box plot respectively. Any instance with an OD score greater than the cutoff is considered an outlier and removed.

In the preliminary analysis on the entire input data the method identified four outliers from the female subjects and two from the male subjects, as shown in the charts of Figure 2. For the performance evaluation with cross-validation, at each fold the outlier detection model (iForest) and the cutoff threshold are computed on the training data and are applied to both training and test sets.

### 3.3. Apparent Brain Age Model

Several studies have adopted machine learning models for the subject's age estimation based on MRI scans of the brain. This is considered to be an estimation of the biological age of the brain and its deviation from the chronological age of the subject can be indicative of an acceleration of the ageing process, including a pathological grey matter atrophy in addition to the normal decline. Previous approaches have considered the estimation of the subject's age from the overall morphology of the brain.

The proposed Apparent Brain Age (ABA) is an estimation of the subject's age based on morphological brain structures that are particularly affected by an accelerated decline induced by a specific pathology, the Alzheimer's disease (AD) in this work.

ABA does not attempt to estimate the subject's age from the whole brain morphology in order to maximise the quality of the regression model. On the contrary the quality of the regression model is not to be considered a useful performance metric for the target classification task. The proposed ABA approach adopts an inductive bias in the regression task from the second and more important classification task. The rationale is to estimate the brain age from healthy (CN) subjects with a learning bias toward those input features that are mostly affected by AD. The ABA regression model is expected to provide a biased estimation of the overall brain age: ABA for subjects affected by AD is expected to be more overestimated than it would be if the entire brain morphology is used. As a consequence, ABA is expected to have a better predictive power for the specific pathology, improving the accuracy of the classification task as well as its specificity. Nevertheless, the regression model is also analysed in terms of the correlation coefficient (*r*) and the Mean Absolute Error (*MAE*) to provide validation and potential insights of the adopted approach.

There are three key machine learning tasks in the data workflow to infer and use ABA for the classification task: the input feature selection process, the inference of a regression model to estimate the brain age and the inference of a predictive model for the classification of AD.

In order to preserve the explainability of the entire workflow from the semantically meaningful input space to the classification output, ABA is inferred with a linear regression model in combination with an aggressive feature selection technique. The objective is to identify the simplest and most explainable model to achieve a prediction accuracy comparable or superior to baseline methods selected from the state of the art approaches based on known morphological structures of the brain.

Feature selection is an NP-hard problem and an exhaustive search of the globally optimal subset of features is not feasible. Among various heuristic methods (Sarica et al., 2014b; Spedding et al., 2015) that can be adopted to find locally optimal solutions to this problem, a wrapper feature selection approach is preferred for its simplicity and straightforward interpretation. Wrapper methods typically adopt an iterative and incremental strategy for the selection of a feature subset (exploration of the input subspaces) that is wrapped around the model inference process. The estimation of the model performance drives the incremental selection of features that are strictly useful.

Typical search techniques for wrapper methods (i.e., backward, forward, and stepwise searches) are applied before the model is created, however these search techniques provide a sequence of discrete decisions that cannot be undone: features are either retained or discarded in a greedy approach. To mitigate this problem a shrinkage method for the inference algorithm can be used in combination to the wrapper method to apply an additional constraint to the regression model inference process by means of an embedded feature selection technique. The adopted shrinkage method for the linear regression model is the Least Absolute Shrinkage and Selection Operator (LASSO) (Tibshirani, 1996), which constraints the sum of the coefficients to be less than a threshold. This constraint acts as a fine grained selection mechanism in addition to the coarse grained mechanism of the wrapper method. LASSO is acting as an embedded feature selection method. The synergistic combination of the two feature selection mechanisms, wrapper and embedded, provides a good trade off between a biased exploration of the large search space and the refinement of local solutions. LASSO provides also a number of other advantages, including reducing the risk of overfitting the training data and, most importantly in this case, reducing the number of explanatory variables that provide minimal contribution to the model, which is consistent to the overall explainability objective.

The linear regression equation for ABA is given by

(2) $$\begin{array}{r} aba=a_{0}+\overset{k}{\underset{i=1}{\sum }}a_{i}\cdot f_{i}, \end{array}$$

where *k* is the number of selected features *f*_*i*_ ∈ *F* and {*a*_*i*_} (0 ≤ *i* ≤ *k*) is the set of the *k* + 1 linear coefficients.

The next section describes the adopted wrapper method, which is a forward feature selection technique based on the predictive power of the individual input features. This method is specifically introduced to provide an inductive bias toward input features highly predictive of AD and determines the specificity of ABA.

### 3.4. Biased Forward Feature Selection

A Forward Feature Selection (FFS) method is applied to identify a subset of features that are biased toward the classification of AD. The method requires a ranking system to sort the input feature and proceed with a sequential iterative order to the evaluation of each feature for its inclusion or exclusion. In a preliminary analysis, various feature scoring techniques have been considered and compared. Some may, in principle, provide a better performance at the cost of additional complexity. However, due to the local refinement of the solution provided by the embedded method, the wrapper method is not required to be particularly accurate; it is used to provide a general direction in the global search strategy.

Thus, a simple and sufficiently effective technique is preferred to minimise the computation time. The input features are ranked in decreasing order of the absolute coefficient of the point-biserial correlation with respect to the binary classification variable. Features with high absolute correlation to the classification task are considered for inclusion in the feature subset before features with lower absolute correlation.

An iterative forward selection technique is applied to the ordered features. Each candidate feature subspace is used to build a LASSO model and to estimate ABA. Age and ABA are then used to infer and test a logistic regression model for the classification task.

The feature selection process is performed at each fold of the overall cross-validation method to ensure good generalisability of the results. The feature selection process itself is an optimisation procedure based on cross-validation, where the partitions for training and test sets are generated from the training set of the external overall cross-validation.

### 3.5. Classification Model Interpretation and Evaluation

The adopted binary classification method (AD vs. CN) is a logistic regression model based on two inputs, the subject's chronological age (*age*) and the estimated brain age (*ABA*). The model is a linear decision boundary between the two classes in the two dimensional space, which can be easily visualised and interpreted. The difference between ABA and age is referred to as Age Deviation Score (ADS), where *ADS* = *ABA* − *age*. In alternative to ABA, the age deviation is often used for visualisation and interpretation.

The linear boundary in the logistic regression for AD classification is given by the inequality

(3) $$\begin{array}{r} c_{0}+c_{1}\cdot age+c_{2}\cdot aba<0. \end{array}$$

Considering equation (2), the classification rule (3) can be expressed directly in terms of the input features according to

(4) $$\begin{array}{r} c_{0}+c_{1}\cdot age+c_{2}\cdot \left(a_{0}+\overset{k}{\underset{i=1}{\sum }}a_{i}\cdot f_{i}\right)<0. \end{array}$$

Assuming *c*_2_ < 0 and (*c*_0_ + *c*_1_ · *age* + *c*_2_ · *a*_2_) < 0, equation 4 can be expressed as:

(5) $$\begin{array}{r} \overset{k}{\underset{i=1}{\sum }}-\frac{c_{2}\cdot a_{i}\cdot f_{i}}{c_{0}+c_{1}\cdot age+c_{2}\cdot a_{0}}>1. \end{array}$$

The score *s*_*i*_ of a feature *f*_*i*_ is defined as its contribution to the classification inequality of (5) and is given by the following equation. A negative or low score indicates the absence or a low level of atrophy due to the neurodegeneration process; while a positive and high value of the score may indicate the presence of abnormal atrophy with the feature contributing toward an AD classification.

(6) $$\begin{array}{r} s_{i}=-\frac{c_{2}\cdot a_{i}\cdot f_{i}}{c_{0}+c_{1}\cdot age+c_{2}\cdot a_{0}} \end{array}$$

The condition in equation (3) for AD classification can be expressed as the sum of the feature scores and can be used to explain the classification prediction in terms of individual features.

(7) $$\begin{array}{r} \overset{k}{\underset{i=1}{\sum }}s_{i}>1 \end{array}$$

#### 3.5.1. Model Evaluation

The main method used to evaluate the performance of the model is the estimation of the classification accuracy by means of a 10-fold cross-validation. At each fold the input data is systematically split into two disjoint sets, training set and test set. The model is trained and built on the training set and tested on the test set. At the end of the 10 folds, the test results are aggregated to compute the estimation of the accuracy. This method ensures that the data points in the test set are not used in training the model, while allowing to compute the accuracy on the entire input data, providing a more robust estimation. For this reason cross-validation is typically preferred over the simpler and less computationally demanding hold-out method. To verify the robustness (low variance) of the performance evaluation, a 10-time repeated 10-fold cross-validation is carried out.

Since the cross-validation method does not provide a single model, a final model for visualisation in the experimental analysis and eventually for deployment in a real-world scenario, is trained on all available input data: this final model is also included in some visualisation, though it is not used for the performance evaluation. In addition, a hold-out method is used to produce a single model that can be used for the visualisation of test cases and the presentation of the explainability offered by the approach.

## 4. Experimental Analysis and Results

For a comparative analysis of the proposed method, two machine learning algorithms, SVM and DNN, were selected because of their ability to process a high-dimensional input space as well as dealing with outliers. A preliminary analysis was used to tune some hyper-parameters and to achieve results comparable to the state of the art for this problem. A linear two-class SVM model with regularisation parameter *c* = 1, is built on all input feature *F* and the *age* for the binary classification task. The DNN architecture has an input layer with a number of units equal to the number of input feature *F* plus one for *age*. Three hidden fully connected layers have about half the number of units with respect to the previous layer (200, 100, 50) and adopt a ReLU activation function. These dense layers are interleaved by dropout (30%) layers for regularisation. The output layer has one unit with a sigmoid activation function.

Three baseline methods (B1, B2, B3) are used. The SVM model is build on the ADNI data only (B1) and on both ADNI and IXI data (B2). The DNN was trained on ADNI and IXI data (B3). In both cases the input data are preprocessed in the same way as in the proposed method.

The complete proposed method M6 includes feature selection, ABA regression and logistic regression for the classification. The method is also tested in two partial configurations, M4 and M5, to provide an indication of the relative importance of some components. The models for these two methods are trained without feature selection: the ABA regression model is inferred on the full set *F* of input feature, similarly to the baseline methods. The method M4 is trained only on ADNI data and the method M5 on the complete input data set including both ADNI and IXI.

The performance analysis of the methods is carried out with a 10-time repeated 10-fold cross-validation method.

### 4.1. Feature Selection

During each cross-validation run, several models are generated, one for each fold. In the proposed method M6, the biased FFS component identifies a subset *F*_1_ of the entire input feature set *F* to be used as input to the LASSO regression inference algorithm. Some of the features in a subset *F*_1_ may be highly correlated and the LASSO regularisation mechanism helps to eliminate redundant, noisy or not sufficiently relevant ones: the ABA model is based on a further reduced subset *F*_2_, with *F*_2_ ⊆ *F*_1_ ⊆ *F*.

While the feature subset *F*_2_ is desired to be minimal and effective for the classification task, according to the minimum description length (MDL) principle (Rissanen, 1978) (aka Occam's razor), the feature subset *F*_1_ can be more informative. The difference is that *F*_2_ helps making the best classification decision according to the model inferred by the given training data, while the set *F*_1_ contains richer and more exhaustive information useful to inform a domain expert.

The average number of features in *F*_1_ over all the models is 14 (8 ≤ |*F*_1_| ≤ 22) in the female group and 16 (10 ≤ |*F*_1_| ≤ 25) in the male group. These feature sets are analysed in the reminder of this section.

The average number of features in *F*_2_ over all the models is 12 in each gender group, with 5 ≤ |*F*_2_| ≤ 20 in female subjects and 4 ≤ |*F*_2_| ≤ 25 in male subjects. These sets are used for the analysis of the feature scores in section 4.3.

The feature subsets *F*_1_ provide an opportunity to learn about the relative importance of each individual feature for the specific classification task. Each ROI can be evaluated in terms of the number of its occurrences in the feature subsets. The result is shown in Table 2. Many of the top regions are related to the hippocampus and its substructures (Schröder and Pantel, 2016), whose atrophy is a trait of AD. Other frequently selected features have also been linked to AD and include the medial temporal lobe (Berron et al., 2020), the amygdala (Poulin et al., 2011), the hippocampus-amygdala transition area (HATA), the entorhinal cortex (Latha Velayudhan et al., 2013), the medial occipitotemporal (fusiform) gyrus (Convit et al., 2000), and the cortical areas around the superior temporal sulcus (bankssts) (Wang et al., 2009).

**Table 2:** ROIs selected over all folds of all cross-validation trials for both genders.

	**F&M**			**F**			**M**		
**ROI**	**LH&RH**	**LH**	**RH**	**LH&RH**	**LH**	**RH**	**LH&RH**	**LH**	**RH**
Entorhinal_thickness	**76%**	**97%**	**54%**	**70%**	**96%**	44%	**81%**	**98%**	**64%**
Whole_hippocampus	**55%**	**100%**	9%	**56%**	**100%**	12%	**53%**	**100%**	6%
Middletemporal_thickness	**52%**	**61%**	43%	**55%**	34%	**76%**	49%	**88%**	10%
Subiculum	44%	39%	49%	**50%**	**66%**	34%	38%	12%	**64%**
CA1	35%	45%	24%	23%	32%	14%	46%	**58%**	34%
Molecular_layer_HP	34%	**67%**	1%	17%	34%	0%	**51%**	**100%**	2%
Amygdala	37%	16%	**58%**	22%	16%	28%	**52%**	16%	**88%**
Hippocampal_tail	25%	31%	19%	19%	20%	18%	31%	42%	20%
Presubiculum	25%	20%	30%	25%	12%	38%	25%	28%	22%
HATA	13%	9%	17%	20%	14%	26%	6%	4%	8%
GC_ML_DG	12%	16%	7%	4%	0%	8%	19%	32%	6%
Fusiform_thickness	11%	15%	7%	4%	4%	4%	18%	26%	10%
Bankssts_thickness	11%	17%	4%	18%	30%	6%	3%	4%	2%
Middletemporal_volume	11%	13%	8%	14%	18%	10%	7%	8%	6%
Inf_Lat_Vent	10%	13%	7%	0%	0%	0%	20%	26%	14%
Inferiortemporal_thickness	10%	8%	12%	9%	10%	8%	11%	6%	16%
CA3	9%	10%	8%	5%	4%	6%	13%	16%	10%
CA4	9%	6%	11%	10%	6%	14%	7%	6%	8%

*Only features selected at least in 10% of the models either for the Left Hemisphere (LH) or the Right Hemisphere (RH) are included. ROIs are listed in decreasing order of the total frequency in all groups and both hemispheres. (Frequencies greater than or equal to 50% are in bold)*.

The effectiveness of the feature selection method is confirmed by the automatic identification of those regions involved in the most important and earliest signs of AD (Braak et al., 1993), i.e., the substructures of the medial temporal lobe, including the entorhinal cortex, the hippocampus and the amygdala.

### 4.2. Predictive Performance

**Figure 3:**
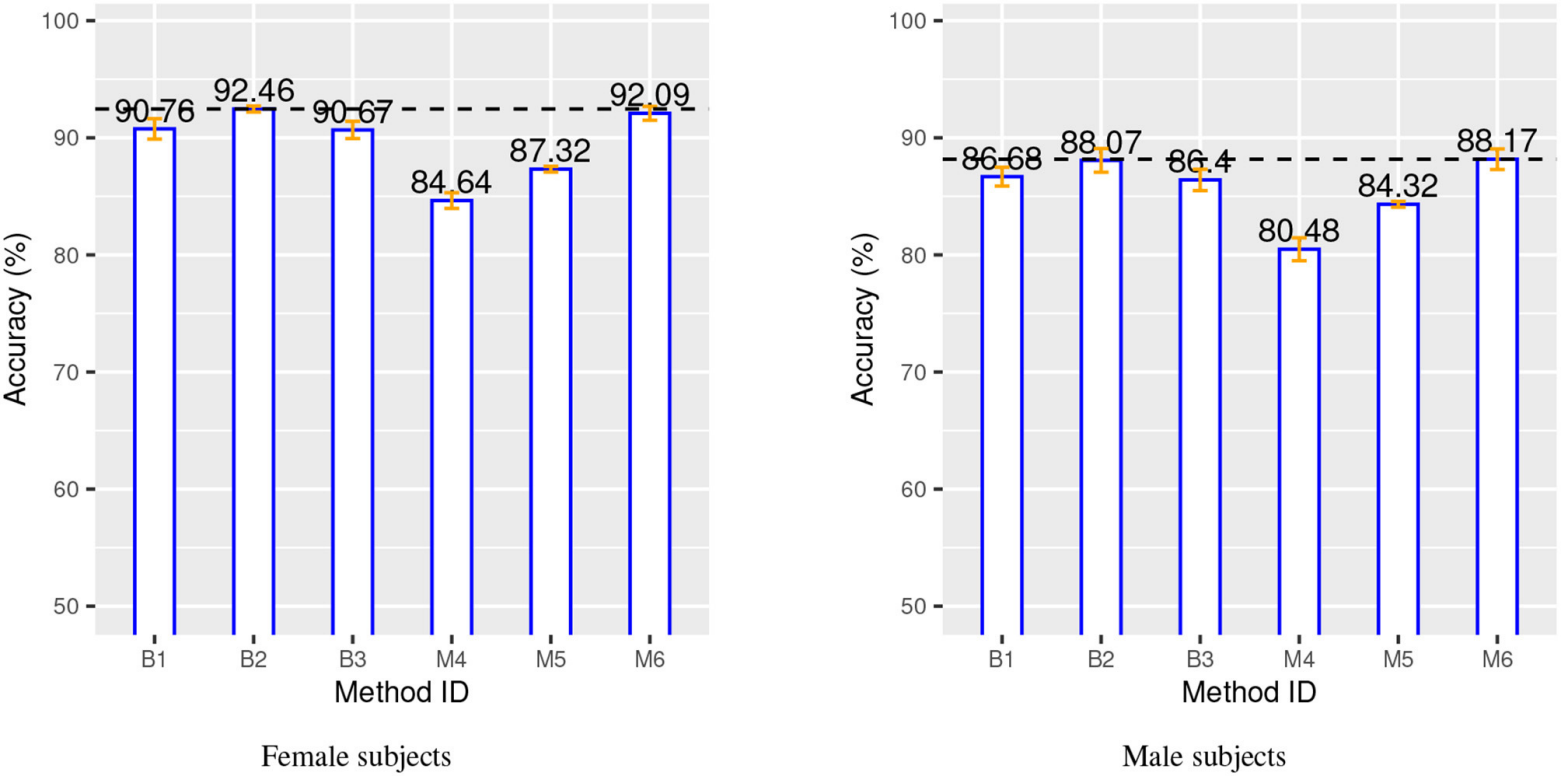
Classification accuracy for both gender groups over 10-time repeated 10-fold cross-validation. Blue bars, orange bars and dashed lines represent, respectively, accuracy, standard deviation, and best accuracy.

The main performance analysis is carried out on ADNI and IXI data with a 10-time repeated 10-fold cross-validation method to produce performance indices. Table 3 provides a summary of the important components and configurations as well as the relevant performance indices for these six methods for a comparative analysis. Figure 3 provides a visual comparison of the accuracy for both gender groups.

**Table 3:** Summary of classification results for both gender groups: three baseline methods (B1, B2, B3) vs. the proposed approach (M6) with different configurations (M4, M5) to highlight the contribution of different components.

				**ABA model**		
		**IXI data**			**IXI data**	
						**FFS**
**Method ID**	**B1**	**B2**	**B3**	**M4**	**M5**	**M6**
Data Sources	ADNI	ADNI+IXI	ADNI+IXI	ADNI	ADNI+IXI	ADNI+IXI
Feature Selection	-	-	-	-	-	biased FFS
ABA Regression	-	-	-	LASSO	LASSO	LASSO
Classification Features	F, age	F, age	F, age	ABA, age	ABA, age	ABA, age
Classification	SVM	SVM	DNN	LogReg	LogReg	LogReg
(M) Accuracy % (SD)	86.68 (0.8)	88.07 (1.01)	86.40 (0.91)	80.48 (0.98)	84.32 (0.25)	**88.17** (0.88)
(M) AD Recall % (SD)	-	-		72.74 (2.02)	72.53 (0.49)	78.74 (2.57)
(M) AD Precision % (SD)	-	-		77.34 (1.52)	79.99 (0.71)	85.67 (0.93)
(F) Accuracy % (SD)	90.76 (0.87)	**92.46** (0.26)	90.67 (0.74)	84.64 (0.67)	87.32 (0.25)	92.09 (0.59)
(F) AD Recall % (SD)	-	-	-	69.66 (1.57)	70.21 (0.72)	78.21 (1.96)
(F) AD Precision % (SD)	-	-	-	77.89 (1.11)	76.32 (1.24)	88.27 (0.91)

*For the target classification task the estimated accuracy (average and standard deviation) is reported (highest value in bold). The precision and recall for the classification target group AD is also reported for the proposed method*.

The two charts in Figure 3 show that the SVM method (B1 vs. B2) benefits from the additional CN data record introduced from the IXI source, with a relative improvement of about 1.5% in both gender groups. This is even more evident for the proposed method, M4 vs. M5, with a relative improvement of about 3 − 4% in both gender groups. The classification-biased feature selection method in M6 provides another improvement to the accuracy with an increase of 4% with respect to M5. The precision and the recall for the target class (AD) are significantly improved by the introduction of the goal-conditioned feature selection method.

The results clearly confirm that the proposed method achieves a comparable or superior performance to the complex baseline machine learning methods in spite of its core linear approach. This is achieved thanks to the appropriate design and combination of techniques to explicitly address specific aspects of the learning process in contrast to the implicit and black-box solution provided by SVM and DNN. In particular, the experimental results validate the effectiveness of the novel ABA feature generation method. The inductive bias injected into the feature selection process allows to estimate an “apparent” brain age from a few automatically selected regions of the brain, which are specifically and highly predictive of AD.

**Figure 4:**
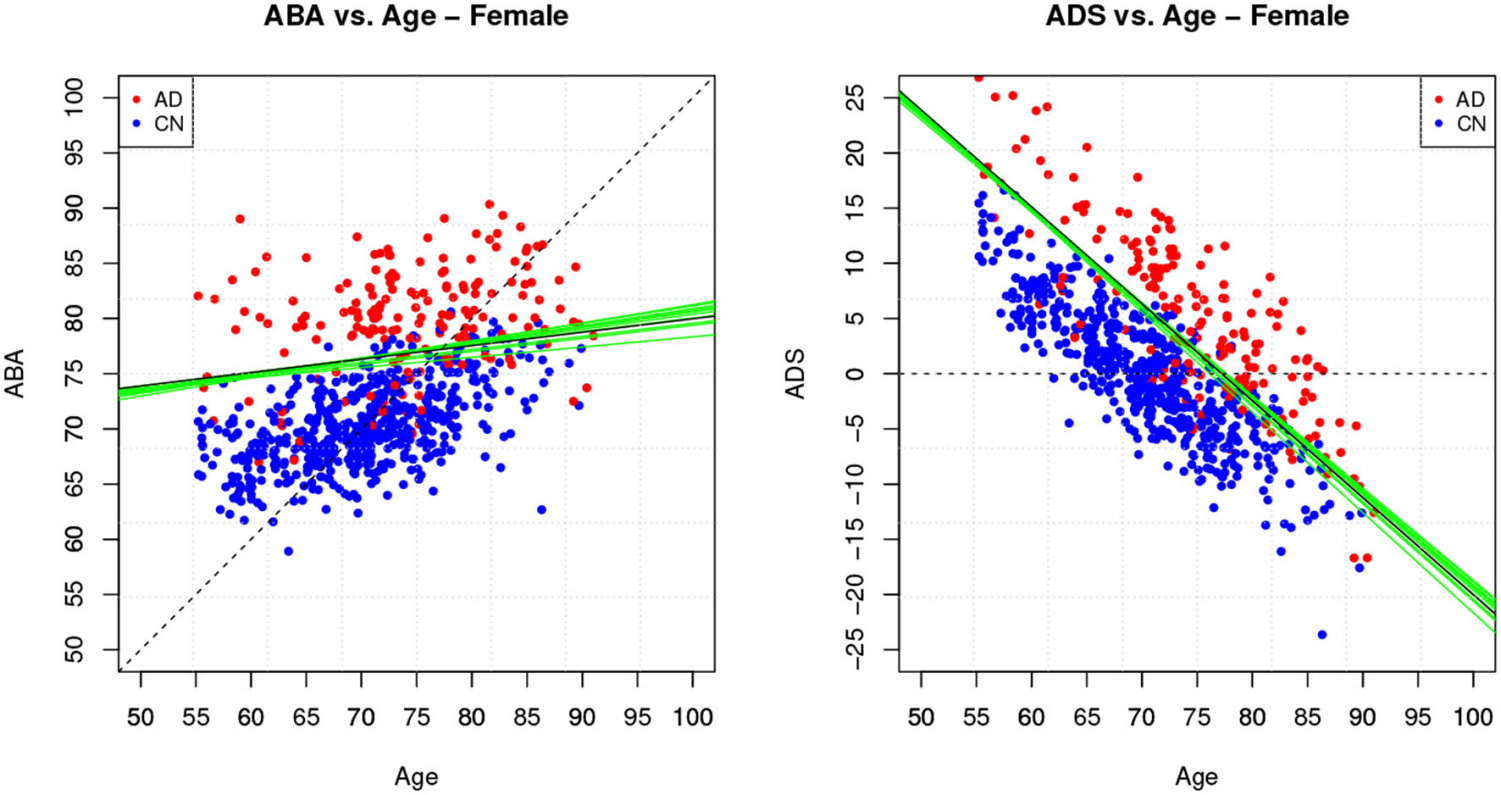
Method M6: Apparent Brain Age vs. chronological age for the female gender group. The plot on the left shows ABA vs. age; the plot on the right shows the ADS vs. age for the same data and with the equivalent boundary line. The ABA values are generated as test set predictions from the folds of a single cross-validation execution. The solid black line is the logistic regression decision boundary of the final model; the green lines are the boundaries in the individual folds of cross-validation; the dashed line is included as reference. The black boundary line is indicative and is obtained from the single final model trained on all data.

**Figure 5:**
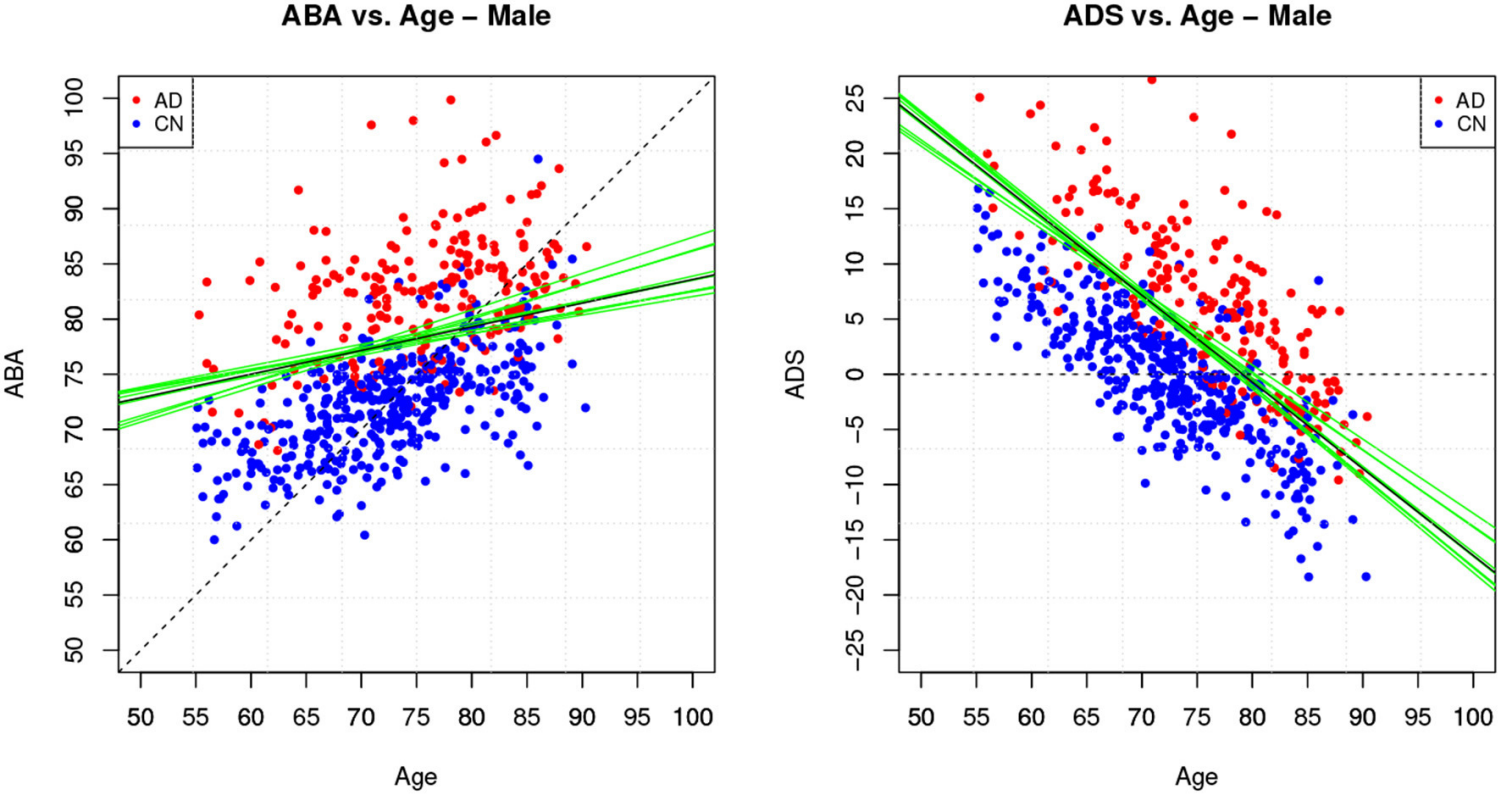
Method M6: Apparent Brain Age vs. chronological age for the male gender group.

**Figure 6:**
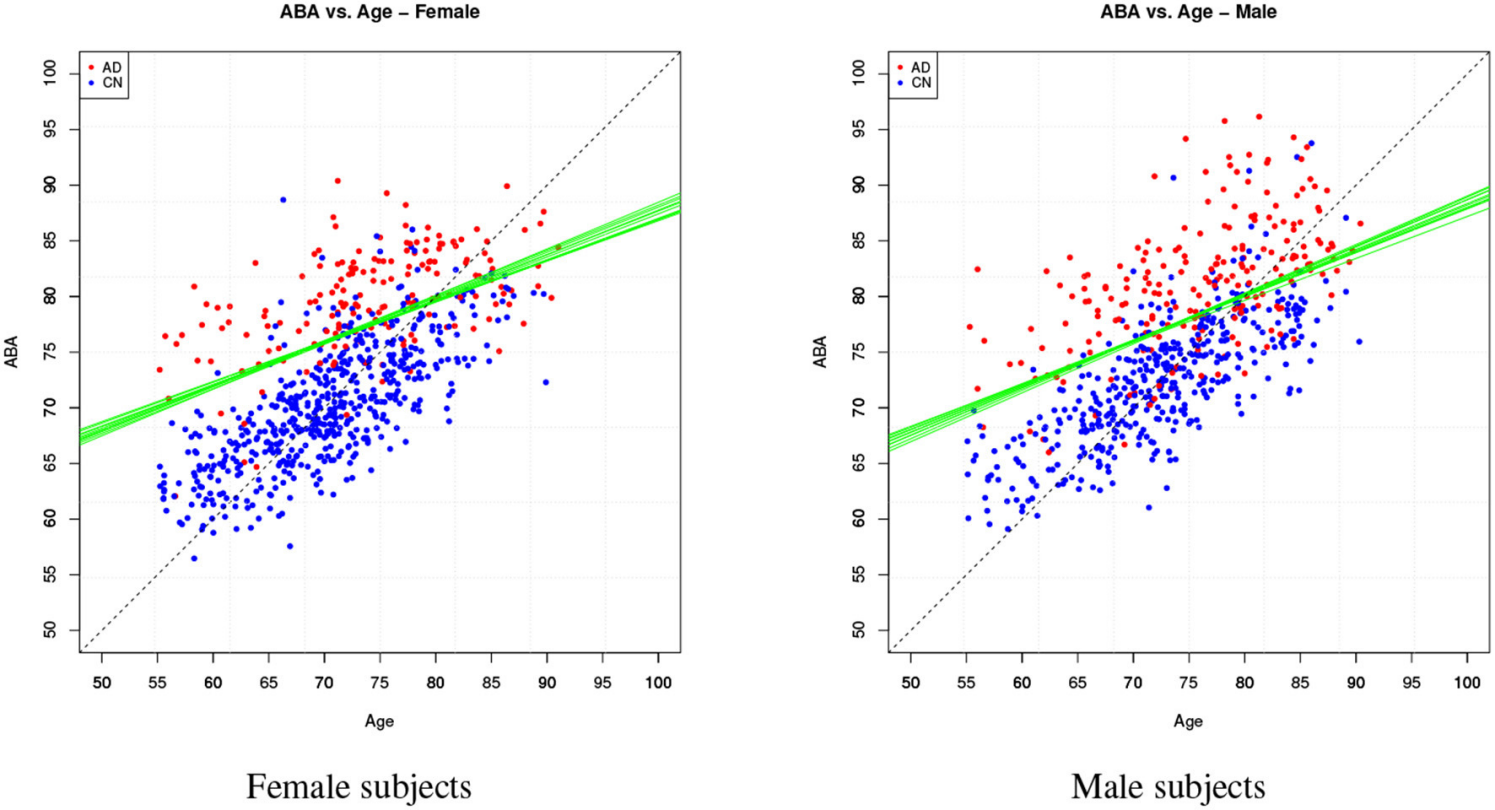
Method M5: ABA vs. Age plots for both gender groups.

Table 4 provides the performance analysis of the regression task. Since the objective is not to maximise the accuracy of the age prediction task, the regression model is not improved by the feature selection process, which is actually decreasing the correlation (*r*) between age and ABA. This is also clearly visible in the comparison of the ABA vs. age plot of the Figures 4, 5 for the method M6, and Figure 6 for the method M5. For example, ABA and age for female CN subjects show a much stronger correlation in Figure 6 (*r* = 0.72) than in Figure 4 (*r* = 0.53). For completeness, the table also includes the performance indices for the holdout method, which is used in the next section.

**Table 4:** Summary of regression results (*MAE*, *r*) for both gender groups: three incremental versions of the proposed methods with different configurations highlight the contribution of different components.

		**MAE**			**r**		
**Data Partition**	**Group**	**M4**	**M5**	**M6**	**M4**	**M5**	**M6**
(M) 10f xval test	CN	3.8	3.91	4.74	0.64	0.75	0.6
(M) 10f xval test	AD	6.08	6.77	8.02	0.54	0.56	0.36
(F) 10f xval test	CN	3.64	3.88	4.71	0.66	0.72	0.53
(F) 10f xval test	AD	6.6	7.11	7.77	0.51	0.53	0.26
(M) holdout training	CN	3.27	3.26	4.94	0.76	0.85	0.55
(M) holdout test	CN	3.9	3.42	4.68	0.61	0.79	0.63
(M) holdout test	AD	5.15	5.76	6.74	0.49	0.51	0.42
(F) holdout training	CN	2.94	2.77	4.83	0.82	0.88	0.51
(F) holdout test	CN	3.64	3.98	4.95	0.64	0.68	0.44
(F) holdout test	AD	6.77	7.53	6.92	0.49	0.36	0.06

*For the ABA regression model the Mean Absolute Error (MAE) and the correlation coefficient (r) are reported. The results refer to the indicated data partition with respect to a performance evaluation method, either the 10-fold cross-validation or the holdout method*.

The linearity of the overall approach enables the investigation and interpretation of the classification predictions directly in the original and semantically meaningful input space. This analysis is carried out in the following section.

Further to the performance analysis reported in this section, two comprehensive tables are provided in the Supplementary Material to summarise the results for the holdout and cross-validation methods for different combinations for training and test sets for the proposed method (M6).

### 4.3. Model Explainability Analysis

The inferred chain of the two linear models is highly descriptive. In this section we use the definition of the feature score in (6) to generate an explanation of the classification decision for some representative test cases.

For the following analysis we use the model generated by a single holdout method with a training-test partition (80–20%) on the female subject group. The outliers detection algorithms has filtered out 7 female subjects and the input data set is reduced to 711 subjects, 570 for the training set and 141 for the test set. The accuracy on the test set is 95.04% (TP = 30, TN = 104, FN = 4, FP = 3).

**Figure 7:**
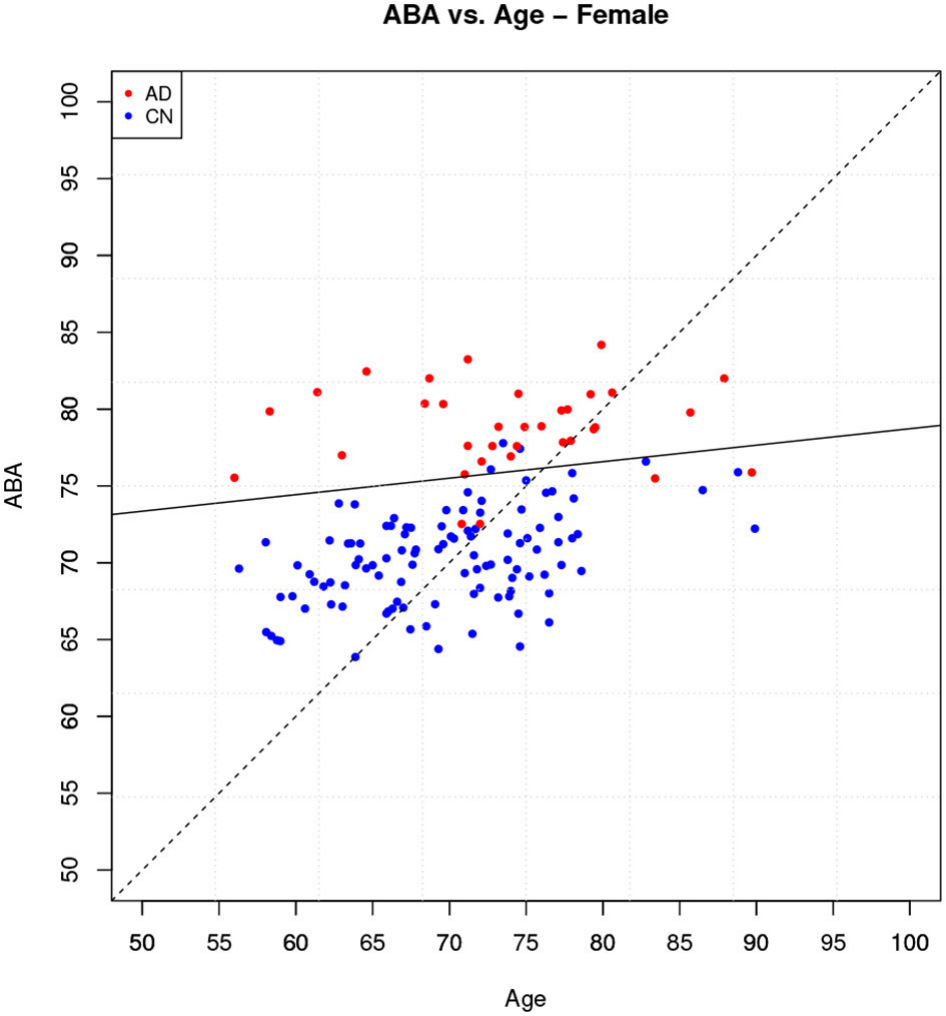
Holdout method: ABA vs. Age plot for the female gender group.

The ABA vs. age plot is shown in Figure 7, which also shows the decision boundary. The 7 incorrect classifications can be easily identified on the plot as being on the “wrong” side of the linear boundary.

**Figure 8:**
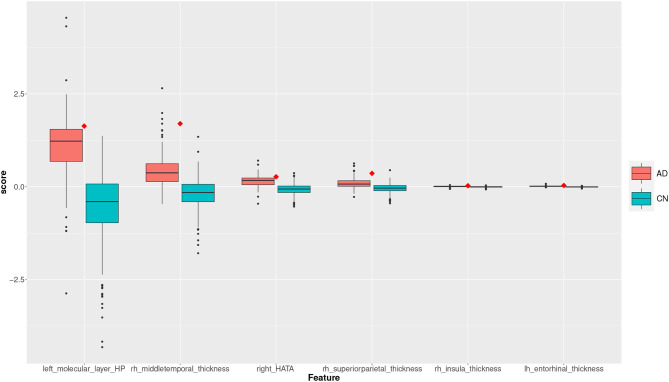
Feature scores of a True Positive (TP) example. The quartiles are generated from the hold-out training data; the example case is selected from the hold-out test data.

**Figure 9:**
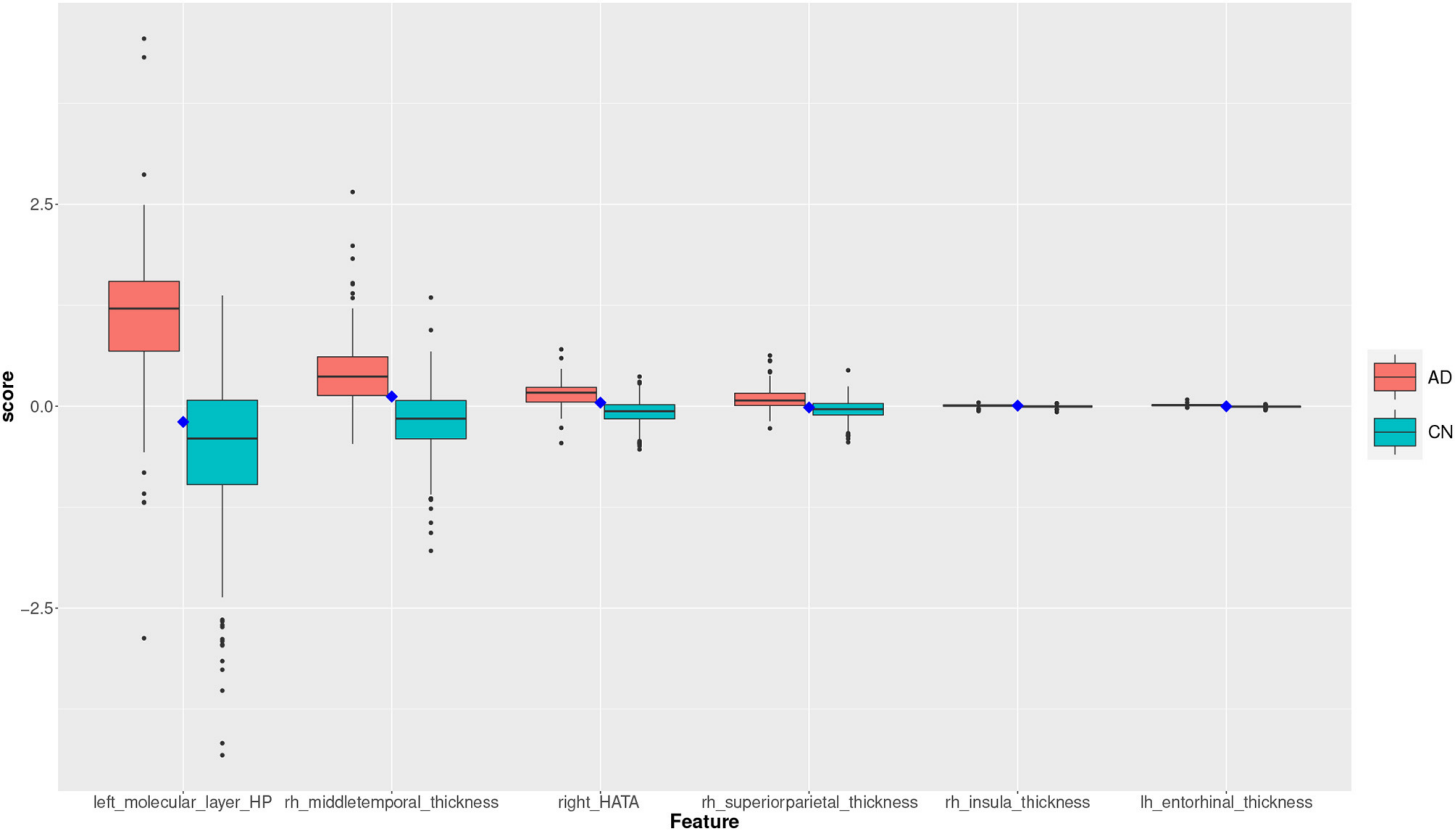
Feature scores of a True Negative (TN) example. The quartiles are generated from the hold-out training data; the example case is selected from the hold-out test data.

Two subjects, a true positive and a true negative, are selected where the feature scores for TP and TN subjects are shown in the plots of the Figures 8, 9. The feature scores for selected FP and FN subjects are shown in the Supplementary Material.

Moreover, the excluded 7 outliers have also been tested with the same holdout model: one was correctly classified, while the other 6 were not. This indicates that the outlier detection method has successfully identified cases for which the classification would be mostly incorrect. The feature scores of one of these incorrectly classified outliers are also shown in the Supplementary Material.

The box plot in Figure 8 shows the feature score distributions for each of the feature in *F*_2_, that is the features used to make the classification decision, through their quartiles: the plot provides a visual representation of statistical data based on minimum, maximum, and the quartiles. A feature score in the lower quartile (Q1) indicates a low contribution toward the classification of AD. The plot includes the quartiles of the AD training records and of the CN training records. A test record, a TP in this case, is superimposed on the plot as single point (diamond symbol) with a colour associated to its actual group (red for AD and blue for CN). In this case, the TP is correctly classified because the top four feature scores are very high, in the highest AD quartile (Q4).

The box plot in Figure 9 shows the feature scores of a TN test record. The scores are mostly in CN Q2/Q3 and none are above the lower AD Q1.

## 5. Independent Data Validation and Discussion

The previous section provided an estimation of the performance of the proposed method with a robust methodology, i.e., 10-fold cross-validation. A further useful validation for comparative purposes is provided by training the model on the subjects of a particular cohort and testing it on an independent cohort of subjects. This allows a direct comparison of the performance with recent studies that have adopted similar test data configurations.

For this purpose, the proposed method (M6) is trained and tested on independent study cohorts and the results are compared with previous works. In this case the analysis is performed on both genders combined for the results to be consistent and comparable to previous works.

In the first case, the model is trained on ADNI1 subjects and tested on ADNI2 subjects achieving an accuracy of 80.17%, which slightly outperforms the accuracy of 79.17% reported in Liu et al. (2018) for an MRI ROI-based deep-learning model trained on ADNI1 and tested on ADNI2.

In the second case, the model is trained on ADNI subjects and tested on AIBL subjects achieving an accuracy of 89.68%, which outperforms the accuracy of 87% reported in Qiu et al. (2020) for an MRI voxel-based deep-learning model trained on ADNI and tested on AIBL.

More detailed results for these two cases are provided in the Supplementary Material.

In both these cases the proposed method achieved similar or better overall accuracy in direct comparison with relevant previous work adopting more complex machine learning algorithms, whose predictions are not easily explainable. In terms of practical applicability of these methods, the proposed feature scores are a powerful descriptive tool that can help to explain and support the classification predictions. Domain experts may find highly desirable to adopt a machine learning approach that not only achieves an excellent predictive performance, but also provides a clear explanation based on semantically meaningful input features (i.e., brain ROIs) that can be directly linked to the diagnosis.

Some limitations of the proposed approach are related to the preprocessing step with FreeSurfer. This step can be quite time consuming with ordinary computing equipment. Moreover, FreeSurfer applies a warping to fit a brain image to a standard template of ROIs: this may introduce volumetric artifacts in the segmentation process.

A limitation of the proposed ABA is that it should not be considered an estimation of the actual age of the subject, as such it may lead to misunderstanding in its interpretation. ABA is meant to under/over estimate the subject age for improving the classification accuracy. ABA is not the estimation of the biological age of the entire brain, rather of a few automatically selected morphological brain regions, which are highly predictive for the specific classification task.

Another intrinsic limitation is the use of linear models in order to provide a good explainability. Explainable non-linear models are an open and interesting direction of research, which may lead to better classification accuracy without compromising the model explainability.

## 6. Conclusions

The Apparent Brain Age (ABA) is an ML-induced biomarker that is specific to a given classification task, rather than being indicative of a general and overall neuroanatomical ageing. In this work the ABA regression model was trained with an inductive bias toward the classification of Alzheimer's disease (AD). In this case, ABA is specialised in the prediction of AD, achieving higher classification accuracy than an age regression model trained without this goal-conditioned inductive bias.

The data workflow adopted in this work was designed specifically to maintain the semantics of the input space throughout the regression and classification tasks. The ABA and the AD classification predicted by this approach are directly linked to a low-dimensional subset of the input feature space, i.e., the Region of Interests (ROI) of the brain image segmentation. The correlation analysis of the semantic input space and the output predictions can generate potentially useful insights on specific test cases as well as, in general, on the induced classification model.

Although the classification model built on ABA and the actual subject age is rather simple in term of model complexity, it has achieved better or comparable AD classification accuracy than state-of-the-art methods such as SVM and DNN, which are not able to provide similar descriptiveness. This was possible by means of a combination of workflow components that explicitly address each aspect of the data modelling task in concertation, while black-box approaches provide a single complex model that addresses them altogether with the downside of learning an internal and intermediate representation that is not intelligible and useful.

The inductive bias adopted in ABA is also expected to provide better specificity in multinominal classification problems. Future work will focus in investigating and validating this hypothesis, for example, with additional data from subjects with a diagnosis of various neurodegenerative diseases and other pathologies that are known to be linked with accelerated ageing of the brain.

## Data Availability Statement

Publicly available datasets were analysed in this study. These datasets can be found at: http://adni.loni.usc.edu/; https://brain-development.org/ixi-dataset/; https://aibl.csiro.au/.

## Author Contributions

GD defined the research problem, the objectives, and the methodology. MR downloaded the original MRI files and pre-processed them with FreeSurfer. AV designed and implemented the general data workflow, the contributed algorithms and the experiments. MS and AA developed additional workflows and code required to carry out some comparative analysis. AV and GD wrote the paper with some contributions from all authors.

## Conflict of Interest

The authors declare that the research was conducted in the absence of any commercial or financial relationships that could be construed as a potential conflict of interest.
