# Supplementary material for: Classification-Biased Apparent Brain Age for the Prediction of Alzheimer's Disease
Source: Front Neurosci. 2021 May 28;15:673120. doi: 10.3389/fnins.2021.673120 (PMC8193935; doi:10.3389/fnins.2021.673120)
Supplement: Supplementary file 1 [file Data_Sheet_1.pdf]

Supplementary Material

These additional tables and figures are provided for completeness.

Table S1. Summary of classification results for each gender separately and both genders combined for the proposed approach (M6) with different datasets. For the target classification task the estimated accuracy and AUC (average and standard deviation) are reported. The F1 score, precision and recall (average and standard deviation) for the classification target group AD are also reported for the proposed method (M6).

Data	Gender	Accuracy % (SD)	AUC (SD)	AD F1 % (SD)	AD Recall % (SD)	AD Precision % (SD)
ADNI	Female	90.18 (1.05)	0.95 (0.0)	83.12 (1.98)	78.71 (2.9)	88.08 (1.38)
	Male	86.72 (0.82)	0.93 (0.01)	82.99 (1.04)	80.66 (1.38)	85.49 (1.43)
	Both	89.34 (0.67)	0.94 (0.0)	84.24 (1.01)	80.8 (1.19)	87.98 (1.03)
ADNI+IXI	Female	92.09 (0.59)	0.95 (0.01)	82.93 (1.34)	78.21 (1.96)	88.27 (0.91)
	Male	88.17 (0.88)	0.94 (0.0)	82.04 (1.54)	78.74 (2.57)	85.67 (0.93)
	Both	89.98 (0.68)	0.95 (0.0)	82.1 (1.09)	78.8 (0.66)	85.71 (2.09)
ADNI+IXI+AIBL	Female	91.12 (0.37)	0.94 (0.01)	77.53 (0.77)	72.26 (1.49)	83.7 (2.04)
	Male	87.3 (0.51)	0.91 (0.0)	76.26 (1.37)	71.27 (2.54)	82.07 (0.62)
	Both	90.2 (0.32)	0.94 (0.0)	78.93 (0.85)	74.6 (1.47)	83.81 (0.7)

Table S2. Summary of holdout classification results for each gender separately and both genders combined for the proposed approach (M6) with different datasets. For the target classification task the estimated accuracy and AUC are reported. The F1 score, precision and recall for the classification target group AD are also reported.

Train Set	Test Set	Gender	Accuracy %	AUC	AD F1 %	AD Recall %	AD Precision %
ADNI1	ADNI2	Female	84.39	0.89	61.73	68.66	88.46
		Male	88.65	0.92	51.76	78.89	97.26
		Both	80.17	0.89	65.24	68.79	83.08
ADNI	AIBL	Female	91.9	0.91	67.5	61.36	75.0
		Male	83.4	0.79	51.22	61.76	43.75
		Both	89.68	0.87	63.75	65.38	62.2

The box plot in figure S1 shows the feature scores of a FP test record. The scores are mostly in AD Q2 and Q3. In particular, the $rh\_middletemporal\_thickness$ is in AD Q3, which is above the median AD score for this region. The scores explain the incorrect classification made by the model. This case would require additional analysis to understand of there is actually a missed diagnosis, or it is classification error due to the natural variability of human brain (we did not consider ICV normalisation in our pre-processing) or due to a low quality (CNR) of the image.

The box plot in figure S2 shows the feature scores of a FN test record. The scores are mostly in CN Q1, Q2 and Q3. There seems to be no indication of abnormal atrophy in the selected features. Similarly, this case would require some additional analysis to identify the causes of the misclassification.

Finally, The box plot in figure S3 shows the feature scores of a misclassified (FN) outlier record. The misclassification can be explained by the fact that the outlier detection has identified this record as 'different' from the majority of the records and further investigation could be carried out to reveal more insights.

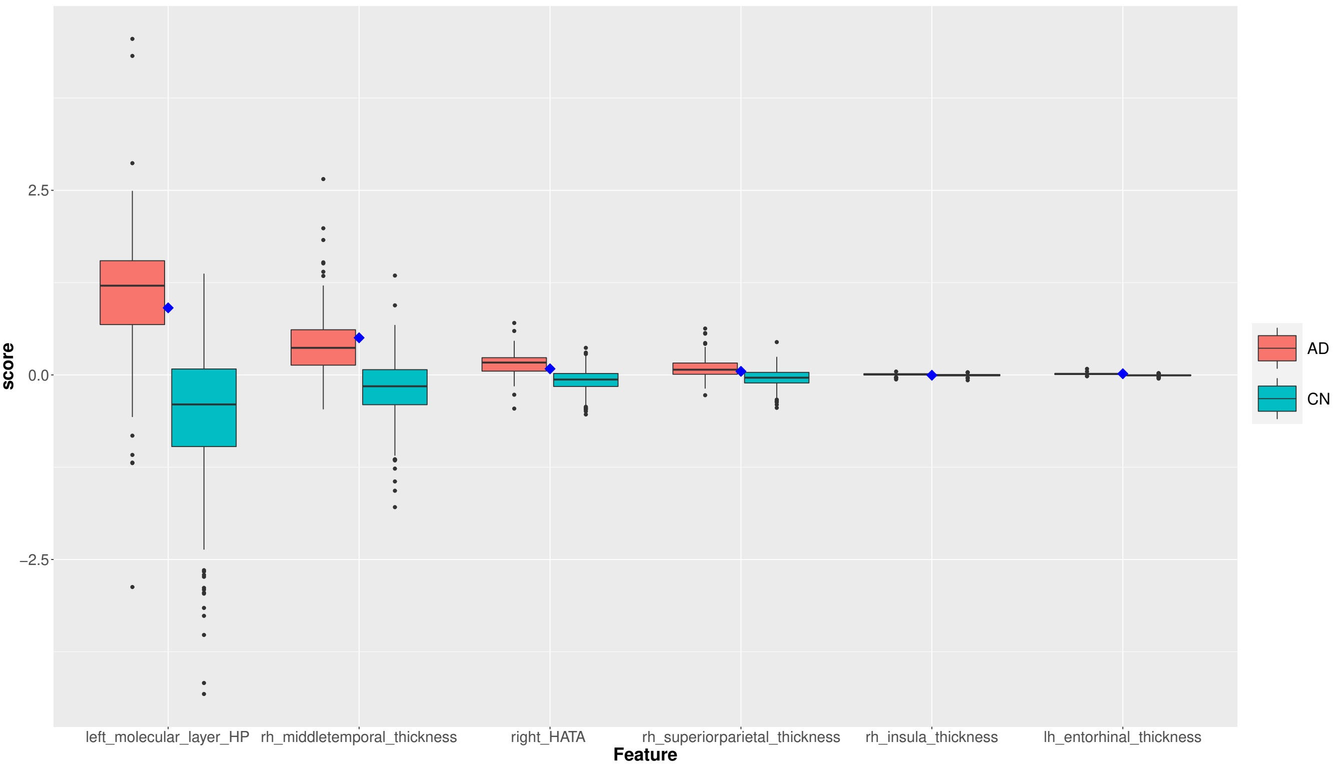

Figure S1. Feature scores of a False Positive (FP) example. The quartiles are generated from the hold-out training data; the example case is selected from the hold-out test data.

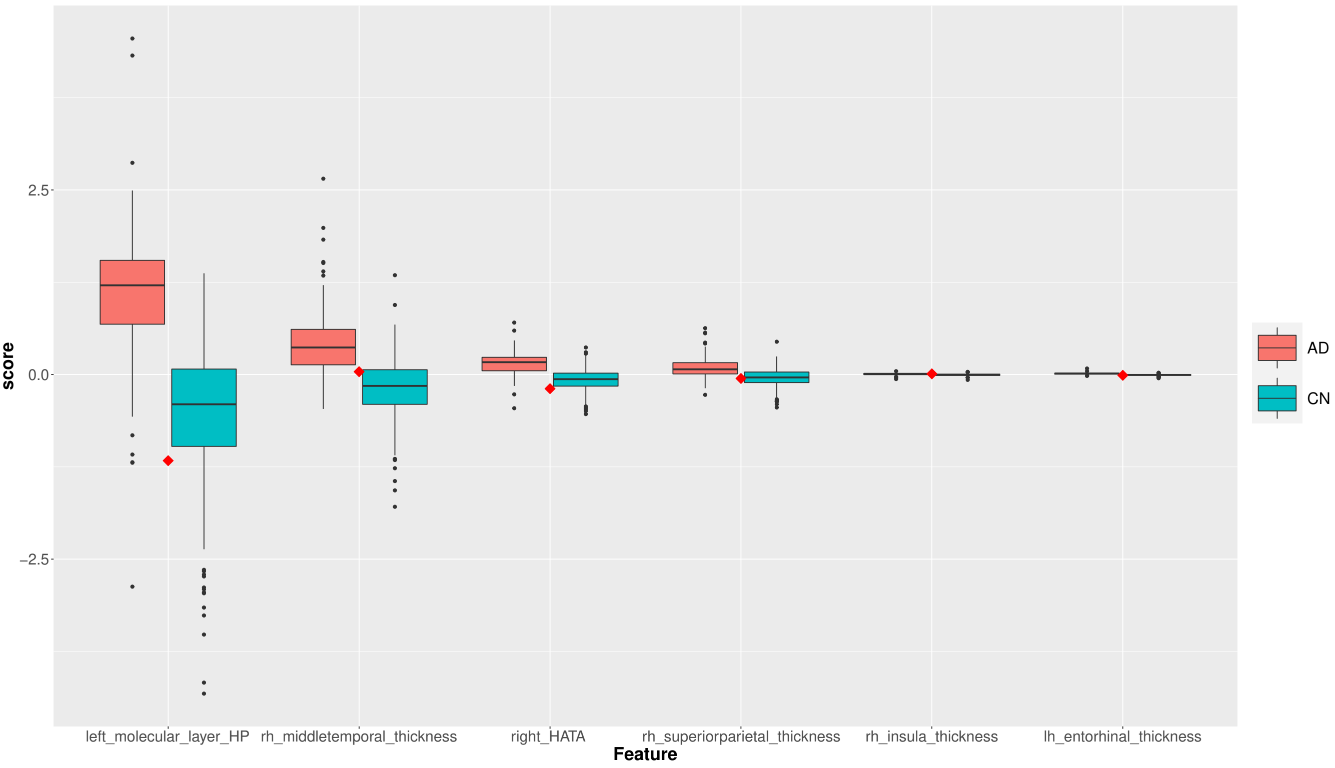

Figure S2. Feature scores of a False Negative (FN) example. The quartiles are generated from the hold-out training data; the example case is selected from the hold-out test data.

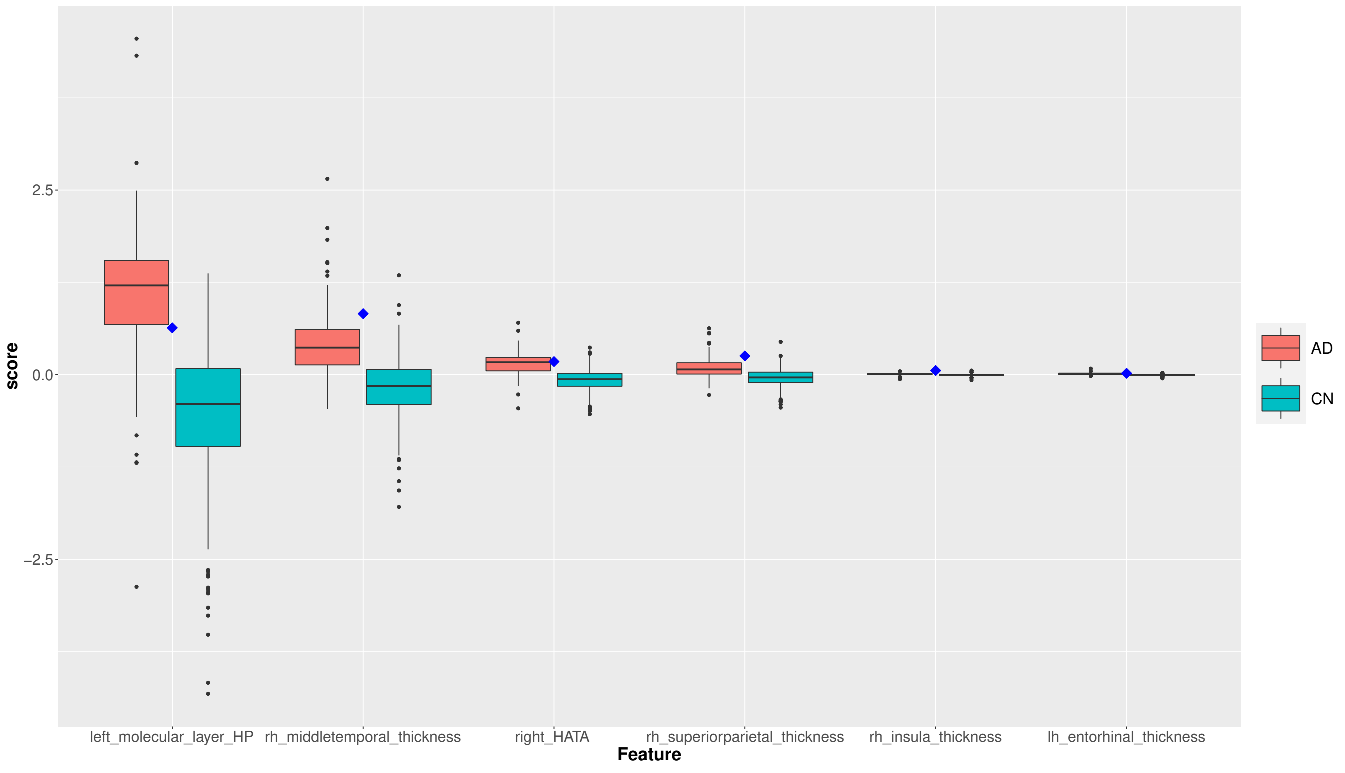

Figure S3. Feature scores of an outlier example. The quartiles are generated from the hold-out training data; the example case is selected from the outlier removed from the hold-out trial. If classified, this would be a FP.
